# Resolution of Sterile Inflammation: Role for Vitamin C

**DOI:** 10.1155/2014/173403

**Published:** 2014-09-09

**Authors:** Bassem M. Mohammed, Bernard J. Fisher, Quoc K. Huynh, Dayanjan S. Wijesinghe, Charles E. Chalfant, Donald F. Brophy, Alpha A. Fowler III, Ramesh Natarajan

**Affiliations:** ^1^Department of Pharmacotherapy and Outcomes Science, Virginia Commonwealth University, Richmond, VA 23298-0533, USA; ^2^Department of Clinical Pharmacy, Faculty of Pharmacy, Cairo University, Cairo 11562, Egypt; ^3^Division of Pulmonary Disease and Critical Care Medicine, Department of Internal Medicine, Virginia Commonwealth University, P.O. Box 980050, Richmond, VA 23298-0050, USA; ^4^Department of Chemistry, Virginia Commonwealth University, Richmond, VA 23298, USA; ^5^Research Service, Hunter Holmes McGuire Veterans Administration Medical Center, Richmond, VA 23249, USA; ^6^Department of Biochemistry and Molecular Biology, Virginia Commonwealth University, Richmond, VA 23298, USA; ^7^The Massey Cancer Center, Richmond, VA 23298, USA

## Abstract

*Introduction*. Macrophage reprogramming is vital for resolution of acute inflammation. Parenteral vitamin C (VitC) attenuates proinflammatory states in murine and human sepsis. However information about the mechanism by which VitC regulates resolution of inflammation is limited. *Methods*. To examine whether physiological levels of VitC modulate resolution of inflammation, we used transgenic mice lacking L-gulono-*γ*-lactone oxidase. VitC sufficient/deficient mice were subjected to a thioglycollate-elicited peritonitis model of sterile inflammation. Some VitC deficient mice received daily parenteral VitC (200 mg/kg) for 3 or 5 days following thioglycollate infusion. Peritoneal macrophages harvested on day 3 or day 5 were examined for intracellular VitC levels, pro- and anti-inflammatory protein and lipid mediators, mitochondrial function, and response to lipopolysaccharide (LPS). The THP-1 cell line was used to determine the modulatory activities of VitC in activated human macrophages. *Results*. VitC deficiency significantly delayed resolution of inflammation and generated an exaggerated proinflammatory response to *in vitro* LPS stimulation. VitC sufficiency and *in vivo* VitC supplementation restored macrophage phenotype and function in VitC deficient mice. VitC loading of THP-1 macrophages attenuated LPS-induced proinflammatory responses. *Conclusion*. VitC sufficiency favorably modulates macrophage function. *In vivo* or *in vitro* VitC supplementation restores macrophage phenotype and function leading to timely resolution of inflammation.

## 1. Introduction

Resolution of inflammation typically follows an ordered series of events orchestrated by different cell types [1]. During the early stages of inflammation, leukocytes such as polymorphonuclear neutrophils (PMN) are the first immune cells to arrive at the site of inflammation. PMN are recruited by gradients of proinflammatory signals and usually reach peak numbers within 24–48 hrs. PMN have short half-lives and are normally cleared from sites of inflammation by undergoing apoptosis [2]. Mobilized monocyte-derived macrophages extravasate to inflammatory tissue sites and clear apoptotic PMN in a nonphlogistic fashion by the process of efferocytosis. Apoptotic PMN release “find-me” signals that are sensed by extravasated macrophages [3]. Following phagocytosis, apoptotic PMN provides resolution cues to macrophages by evoking distinct signaling events that block release of proinflammatory mediators thus allowing further engulfment of apoptotic cells. Mantovani et al. and Fleming and Mosser note that mobilized macrophages are divided into three groups based upon their activation states [4, 5]. These include the M1, M2, and the recently described regulatory macrophages (M_res_). M1 macrophages, classically referred to as activated macrophages, secrete proinflammatory factors that mediate host defense against invading pathogens. M2 macrophages, termed alternatively activated macrophages, are considered to be anti-inflammatory [6, 7]. Finally, M_res_ macrophages secrete considerable amounts of anti-inflammatory cytokines that prevent inflammatory and autoimmune pathology [8, 9]. M_res_ macrophages also secrete various lipid mediators that play critical roles in resolution of inflammation (see below). Extensive new research has identified expression markers or phenotypic signatures for the various macrophage activation states in mice. They include gene expression changes in IL-1*β*, TNF*α*, and iNOS for classical activation and arginase-1 (Arg1), chitinase 3-like 3 (YM-1), and TGF*β* for the alternatively activated macrophages [7].

Resolution of inflammation and restoration of normal tissue function prevent the development of “complications” of excessive inflammation, a process referred to as catabasis [10]. Catabasis is driven by synthesis and release of proresolution lipid mediators such as resolvins, protectins, and lipoxins [11]. Lipoxins [12] and protectins [13] are synthesized by lipoxygenase enzymes (such as 15-lipoxygenase (15-Lox)). Resolvins are derived from omega-3 polyunsaturated fatty acids such as docosahexaenoic acid and eicosapentaenoic acid [14]. They are products of metabolism involving 15-Lox and cyclooxygenase. Russell and Schwarze have reviewed the proresolution effects of proresolution mediators in a variety of inflammatory states [15]. However their regulation by vitamin C (VitC, ascorbic acid, AscA) has yet to be examined.

VitC readily functions as one or two electron-reducing agents for many oxidants and serves as a primary chemical antioxidant in most cell types. It modulates complex biochemical pathways that form an essential part of normal metabolism of immune cells [16]. Intracellular levels of VitC in cells differ significantly from circulating plasma levels. In particular, VitC accumulates in millimolar quantities in immune cells such as PMN and macrophages in which intracellular VitC concentrations are typically 40–60 fold higher than that present in circulation [17, 18]. In PMN, Vissers and Wilkie showed that intracellular VitC levels regulate neutrophil apoptosis [19]. Further, VitC contributes to the antioxidant defenses as well as normal PMN and macrophage function. Oberritter and colleagues showed that intracellular concentrations of VitC in macrophages are in the low millimolar range in freshly prepared peritoneal macrophages and* in vivo* or* in vitro* activation of peritoneal macrophages results in a significant decline in their VitC content [20]. Li et al. found that VitC deficiency worsens the inflammatory response following infection with the influenza virus [21]. Moreover, mice deficient in VitC generate excessive proinflammatory responses upon infection with the virulent bacterium* Klebsiella pneumonia* [22]. In humans, VitC levels are significantly reduced in critically ill patients and specifically in patients with poorly resolving proinflammatory states (e.g., sepsis, systemic inflammatory response syndrome) [23, 24]. Several studies performed in septic patients have found that plasma VitC levels correlate inversely with the incidence of organ failure and directly with survival [25, 26]. We recently showed that VitC attenuates inflammation and normalizes PMN function in septic mice [27, 28]. We further showed that parenteral VitC attenuates proinflammatory biomarkers and reduces mortality in human sepsis [29]. However information is limited regarding the mechanism by which VitC regulates the progression and eventual resolution of inflammatory states.

In the current study we examined the progression and resolution of inflammation using a murine thioglycollate (TG-)elicited peritonitis model in VitC sufficient and deficient mice. While humans lack L-gulono-*γ*-lactone oxidase (Gulo), the final enzyme in the biosynthesis pathway of VitC [30], mice express functional Gulo, resulting in cells and tissues generally maintaining high levels of VitC thereby complicating the translatability of VitC studies in mice, to humans. In order to establish the studies more relevant to humans, TG-induced peritonitis was performed in transgenic mice lacking Gulo (Gulo^−/−^). Our studies reveal that progression and resolution of TG-induced inflammation is significantly delayed in VitC* deficient* mice. In particular, the spatiotemporal profile of pro- and anti-inflammatory mediator production by TG-elicited macrophages was significantly different between the VitC* sufficient* and* deficient* mice. Further, macrophage function and phenotype, as well as the antioxidant capacity of VitC* deficient* macrophages, was significantly impaired by the decline in intracellular VitC levels. Infusion of parenteral VitC as ascorbic acid (AscA) partly restored macrophage phenotype and function in VitC deficient mice.

## 2. Materials and Methods

### 2.1. Animals

Gulo^−/−^ mice were bred in-house from an established homozygous colony as previously described [27]. In order to maintain their plasma VitC levels similar to that observed in humans, VitC sufficient mice were fed ad libitum with regular chow and water supplemented with vitamin C (0.33 g/L) renewed twice per week. Gulo^−/−^ mice were made VitC deficient by reducing VitC supplementation (0.033 g/L) for 1 week, followed by complete removal of dietary VitC for additional 2 weeks. We and others have shown that this reduced supplementation significantly decreases the concentration of VitC in immune cells, plasma, and organs [27, 31, 32].

### 2.2. Thioglycollate Induced Peritonitis and Isolation of Mouse Peritoneal Macrophages

Thioglycollate-mediated peritonitis was established by intraperitoneal (i.p.) injection of 1 mL aged, sterile 3% TG solution to 9–11-week old Gulo^−/−^ mice. Thirty minutes following i.p. challenge, some VitC deficient mice were randomized to receive daily i.p. injection of VitC as AscA (200 mg/kg in saline) for a further 3 or 5 days. Untreated mice received i.p. saline instead of VitC. Mice were euthanized on day 3 or day 5, and the peritoneal cavity lavaged with 7 mL of Hanks' balanced salt solution (HBSS) containing 1% bovine serum albumin (BSA). The lavage was centrifuged and the resulting leukocyte pellet was washed with HBSS and resuspended in RPMI-1640 medium. Cell counts of the peritoneal lavage were performed using a Hemocytometer. Cytochemical staining of peritoneal cells was performed using HARLECO Hemacolor solution (EMD Millipore) [28]. PMNs were then separated from macrophages by adherence to a plastic dish as described previously [28, 33]. Peritoneal macrophages were plated at a density of 2 × 10^6^ cells in 35 mm dishes in growth media (DMEM, 10% FBS). Media were changed after 2 h to remove floating cells prior to experimentation.

### 2.3. Cell Culture

Human acute monocytic leukemia suspension cell line (THP-1) was obtained from ATCC (Manassas, VA). THP-1 cells were maintained in RPMI-1640 medium containing 10% FBS according to the instructions supplied. For induction of macrophages, PMA (100 nM) was added to the medium and cells were seeded at a density of 0.1 × 10^6^ cells/cm^2^ into tissue culture dishes and maintained in a humidified atmosphere of 95% air and 5% CO_2_. Media containing PMA were replaced every 2 days, and experiments started after 5 days in culture, when the cells were phenotypically macrophage [34].

### 2.4. Vitamin C Analysis

Plasma and intracellular VitC levels of peritoneal macrophages seeded onto 35 mm dishes were measured using a fluorescence end-point assay adopted from Vislisel et al. [35]. Plasma was deproteinized as described previously [29]. Briefly, 0.2 mL of cold 20% trichloroacetic acid (TCA) and 0.2 mL of cold 0.2% dithiothreitol (DTT) were added to 0.1 mL of plasma, vortexed for 2 min, and centrifuged (10,000 g, 10 min, 4°C). Supernatants were aliquoted and frozen at −70°C for batch analysis. Peritoneal macrophages were similarly extracted with TCA and DTT and frozen at −70°C for batch analysis. Supernatant or AscA standards were transferred in triplicate to a 96-well plate. Assay buffer containing 1 M sodium acetate, pH 5.5, and 1 mM TEMPOL was added to each well and the plate was incubated for 10 minutes at room temperature. Freshly prepared o-phenylenediamine (OPDA) solution (5.5 mM OPDA in acetate buffer of pH 5.5) was then added. After a further 30 min incubation in the dark, fluorescence was measured at an emission wavelength of 425 nm following excitation at 345 nm and values determined after comparison to a standard curve. Intracellular AscA levels were estimated spectrophotometrically from the standard curve and the intracellular concentrations derived from the measured amount of AscA and the known macrophage cell volume [36].

### 2.5. RNA Isolation and Real-Time Quantitative PCR (QPCR) Analysis

Isolation of total RNA and real-time QPCR analyses were performed as described previously [37]. Briefly total RNA was extracted and purified using QIAshredders and RNeasy columns according to the manufacturer's specifications (Qiagen). Total RNA (1 *μ*g) was reverse-transcribed into cDNA using the High Capacity cDNA Reverse Transcription kit. Complimentary DNA (cDNA) was diluted (1 : 500) and real time QPCR performed using POWER SYBR Green QPCR Master Mix. Primers were designed to anneal to sequences on separate exons or to span two exons. Primers used for QPCR are listed in Table 1. Cycling parameters were 95°C, 10 min, 40 cycles of 95°C, 15 sec, and 60°C, 1 min. A dissociation profile was generated after each run to verify specificity of amplification. All PCR assays were performed in triplicate. No template controls and no reverse transcriptase controls were included. The mRNA expression in macrophages from a “sufficient” mouse or a media well was set to “1.” The mRNA expression of all other samples was compared relative to this sample which was used as the baseline. 18S rRNA was used as housekeeping gene against which all the samples were normalized for differences in the amount of total RNA added to each cDNA reaction and for variation in the reverse transcriptase efficiency among the different cDNA reactions. Automated gene expression analysis was performed using the Comparative Quantitation module of MxPro QPCR Software (Agilent).

### 2.6. Western Blot Analysis

Mouse macrophage and THP-I whole-cell extracts were isolated for western blot analysis as described previously [37]. Proteins were resolved by SDS polyacrylamide gel electrophoresis (4–20%) and electrophoretically transferred to polyvinylidene fluoride membranes (0.2 *μ*m pore size). Immunodetection was performed using chemiluminescent detection with the Renaissance Western Blot Chemiluminescence Reagent Plus (Perkin Elmer Life Sciences Inc., Boston, MA). Blots were stripped using the Restore Western Blot Stripping Buffer (Pierce Biotechnology Inc., Rockford, IL) as described by the manufacturer. Purified rabbit polyclonal antibodies to phospho-NF*κ*B p65 (Ser276, Cell Signaling), NF*κ*B p65 (sc-109, Santa Cruz Biotechnology), iNOS (sc-650, Santa Cruz Biotechnology), and actin (sc-1616, Santa Cruz Biotechnology) were used. Optical densities of antibody-specific bands were determined using Quantity One acquisition and analysis software (Bio-Rad, Hercules, CA).

### 2.7. Flow Cytometry

Mouse peritoneal lavage obtained on day 3 and day 5 from VitC sufficient or deficient mice following induction of TG-induced peritonitis was pelleted by centrifugation at 4°C. Cells were resuspended in FACS buffer containing Fc receptor block (CD16/CD32 eBioscience) for 10 min at 4°C. Aliquots of the suspension were incubated at 4°C for 30 min (in the dark) with fluorescein isothiocyanate (FITC-)conjugated anti-mouse CD45 (eBioscience) and allophycocyanin (APC-)conjugated anti-mouse CD11b (eBioscience). Unstained and single color controls were employed for each experiment. Samples were then fixed with 1% formaldehyde for 20 min at room temperature. All runs were performed on a BD Accuri C6 Flow Cytometer (BD Accuri Cytometers, MI, USA) and analyzed using FlowJo software (Tree Star, Ashland, OR).

### 2.8. Fluorescence Microscopy

Fluorescence microscopy for evaluation of mitochondrial reactive oxygen species (ROS) in macrophages was performed using the cell-permeant probe MitoTracker Red CMXRos as described by the manufacturer. Briefly, macrophages from VitC sufficient or deficient mice were grown on Ibidi 6-channel IbiTreat *μ*-slide VI. Following treatments (H_2_O_2_, 18 hours) culture media were aspirated and cells were fixed in 3.7% paraformaldehyde in PBS for 10 minutes at 4°C. Fluorescence imaging was performed using an Olympus model IX70 inverted phase microscope (Olympus America, Melville, NY) outfitted with an IX-FLA fluorescence observation system equipped with a TRITC filter cube (530–560 nm excitation and 590–650 nm emission, Chroma Technology Corp. Brattleboro, VT) through an Uplan FI objective (40x). Fluorescence images were digitized and captured by a MagnaFire digital camera (Optronics, Goleta, CA).

### 2.9. Lipid Extraction and Analysis

Quantitative analysis of eicosanoids was performed as previously described by us with minor modifications [38–43]. Briefly, peritoneal lavage was clarified by centrifugation and 0.05% BHT and 10 ng of each internal standard added. The internal standards used were (*d*_4_) 8-iso PGF_2*α*,_ (*d*_11_) 5-iso PGF_2*α*_-VI, (*d*_4_) 6k PGF_1*α*_, (*d*_4_) PGF_2*α*_, (*d*_4_) PGE_2_, (*d*_4_) PGD_2_, (*d*_4_) LTB_4_, (*d*_5_) Lipoxin A4, (*d*_5_) Resolvin D2, (*d*_4_) TXB_2_, (*d*_4_) LTC_4_, (*d*_5_) LTD_4_, (*d*_5_) LTE_4_, (*d*_8_) 5-hydroxyeicosatetranoic acid (5HETE), (*d*_8_) 15-hydroxyeicosatetranoic acid (15HETE), (*d*_8_) 14,15 epoxyeicosatrienoic acid, (*d*_8_) arachidonic acid, and (*d*_5_) eicosapentaenoic acid. The samples were mixed by vortexing and subjected to purification via solid phase extraction (SPE) using a 24 port vacuum manifold (Sigma-Aldrich). Strata-X SPE columns (Phenomenex) were washed with methanol (2 mL) and then dH_2_O (2 mL). The samples were applied to the column. Thereafter the sample vials were rinsed with 5% MeOH (2 mL), which was then used to wash the columns. Finally, the eicosanoids were eluted with isopropanol (2 mL). The eluents were then dried under vacuum and reconstituted in LCMS grade 50 : 50 EtOH : dH_2_O (100 *μ*L) for eicosanoid quantitation via UPLC ESI-MS/MS analysis. A 14-minute reversed-phase LC method utilizing a Kinetex C18 column (100 × 2.1 mm, 1.7 *μ*m) and a Shimadzu UPLC was used to separate the eicosanoids at a flow rate of 500 *μ*L/min at 50°C. The column was first equilibrated with 100% Solvent A (acetonitrile : water : formic acid (20 : 80 : 0.02, v/v/v)) for two minutes and then 10 *μ*L of sample was injected. 100% Solvent A was used for the first two minutes of elution. Solvent B (acetonitrile : isopropanol (20 : 80, v/v)) was increased in a linear gradient to 25% Solvent B to 3 minutes, to 30% by 6 minutes, to 55% by 6.1 minutes, to 70% by 10 minutes, and to 100% by 10.1 minutes. 100% Solvent B was held until 13 minutes and then was decreased to 0% by 13.1 minutes and held at 0% until 14 minutes. The eluting eicosanoids were analyzed using a hybrid triple quadrapole linear ion trap mass analyzer (ABSciex 6500 QTRAP) via multiple-reaction monitoring in negative-ion mode. Eicosanoids were monitored using species specific precursor → product MRM pairs. The mass spectrometer parameters used were curtain gas: 30; CAD: High; ion spray voltage: −3500 V; temperature: 300°C; Gas 1: 40; and Gas 2: 60; declustering potential, collision energy, and cell exit potential were optimized per transition.

### 2.10. Statistical Analysis

Statistical analysis was performed using SAS 9.3 and GraphPad Prism 6.0 (GraphPad Software, San Diego, CA, USA). Data are expressed as mean ± SE. Results were compared using one-way ANOVA and the post hoc Tukey test. Statistical significance was confirmed at a *P* value of <0.05.

## 3. Results

### 3.1. VitC Deficiency Alters the Progression of TG-Induced Peritoneal Inflammation

**Figure 1:**
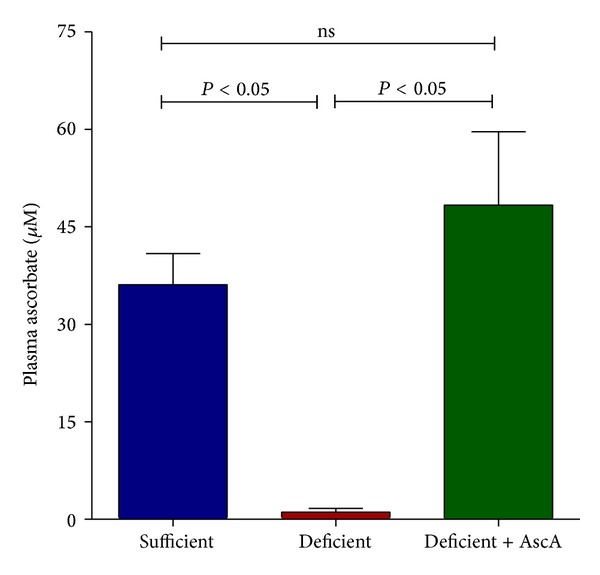
Vitamin C deficiency alters the progression of  TG-induced peritoneal inflammation. Plasma VitC levels were measured in VitC* sufficient* and* deficient* Gulo^−/−^ mice as well as in* deficient* mice treated daily with i.p. AscA for 3 days (*N* = 3–6 mice/group, ns = not significant).

In order to make the Gulo^−/−^ mice VitC deficient, supplementation of water with AscA was withdrawn as described in the Methods section. Within 3 weeks of removal of VitC supplementation, plasma VitC levels of Gulo^−/−^ mice declined significantly (Figure 1). This decline was not associated with deleterious changes in weight or health status in the VitC deficient mice (data not shown). To determine whether VitC deficiency impacts the progression of peritoneal inflammation, VitC sufficient or deficient mice were injected with TG and the progression of inflammation was monitored on days 3 and 5 (as described in Section 2). Some VitC deficient mice were injected i.p. with AscA (200 mg/kg) prior to harvest of peritoneal lavage (see Section 2). Daily i.p. administration of ascorbate for 3 days restored circulating plasma VitC concentrations in these mice to levels observed in the VitC sufficient mice (Figure 1). In all 3 groups, the infiltration of inflammatory cells on day 1 was similar to that observed in wild type mice [44] and was in agreement with our previous observations (Table 2) [28]. As seen in Table 2, there was also no difference in the total number of cells elicited from the peritoneal exudation of day 3 and day 5. However, significant differences in the cellular composition of the lavage were evident on day 3 and day 5 between the 3 groups. In the VitC sufficient mice group, mononuclear cells were the predominant cell type on days 3 and 5 (Table 2). PMN numbers, which peaked on day 1 [28], returned to baseline by days 3 and 5. In contrast, significantly elevated numbers of PMNs persisted in the peritoneal exudates of  VitC deficient mice on days 3 and 5 (Table 2). Infusion of AscA reduced PMN numbers by day 3 with a significant decline in PMN numbers to baseline similar to the VitC sufficient mice by day 5 Table 2.

### 3.2. Spatiotemporal Profiling of Inflammatory Mediators following TG-Induced Peritoneal Inflammation

**Figure 2:**
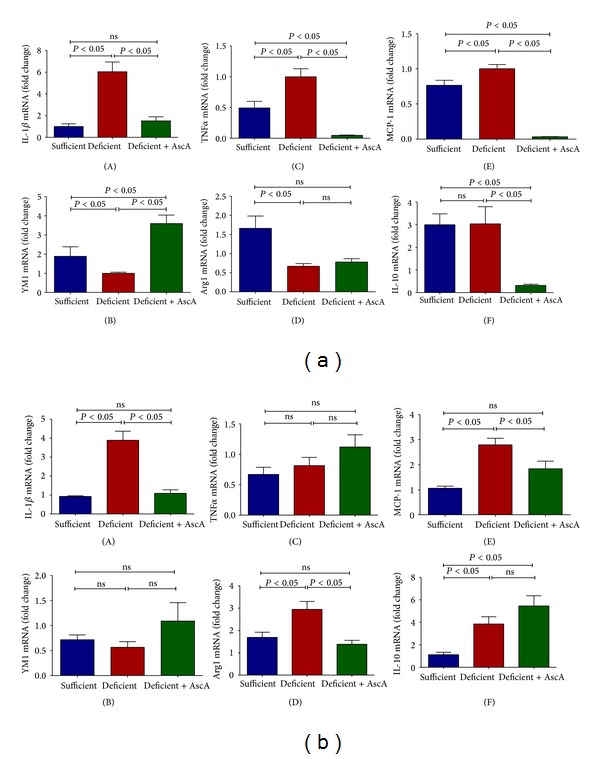
Spatiotemporal profiling of inflammatory mediators following TG-induced peritoneal inflammation. Real time QPCR for IL-1*β*, TNF*α*, MCP-1, YM1, Arg1, and IL-10 mRNA from peritoneal macrophages elicited on day 3 (a) and day 5 (b) following TG-induced peritonitis from VitC sufficient and deficient Gulo^−/−^ mice. Following TG challenge, some VitC deficient mice were randomized to receive daily i.p. injection of VitC as AscA (200 mg/kg in saline) for a further 3 days (day 3, deficient + AscA) or 5 days (day 5, deficient + AscA) (*N* = 6 mice/group, ns = not significant).

We previously observed that TG-elicited PMN from VitC deficient mice (on day 1) demonstrated increased expression of the proinflammatory genes TNF*α* and IL-1*β* [28]. Here we examined the expression of multiple pro- and anti-inflammatory mediators originating from macrophages, the predominant cell type recruited to the inflamed peritoneum on days 3 and 5. As seen in Figure 2, significant differences were evident in the magnitude of pro- and anti-inflammatory mediator expression on days 3 and 5. On day 3, increased expression of the proinflammatory mediators (IL-1*β*, TNF*α*, and MCP-1) was observed in macrophages from VitC deficient mice when compared to macrophages from VitC sufficient mice (Figure 2(a), (A), (C), and (E)). Proinflammatory gene expression was significantly attenuated by i.p. infusion of AscA in the VitC deficient mice (Figure 2(a), (A), (C), and (E)). In contrast, anti-inflammatory gene expression (YM1 and Arg1, but not IL-10) was elevated in macrophages from VitC sufficient mice (Figure 2(a), (B), (D), and (F)). Daily AscA infusion induced YM1 expression in VitC deficient macrophages but failed to restore Arg1 expression. IL-10 expression on the other hand was significantly lowered by AscA infusion on day 3 (Figure 2(a), (B), (D), and (F)).

On day 5 (Figure 2(b)), proinflammatory gene expression remained persistently elevated in macrophages from VitC deficient mice (IL-1*β* and MCP-1) but was attenuated by AscA infusion. In contrast, anti-inflammatory gene expression in VitC deficient macrophages was significantly higher when compared to macrophages from VitC sufficient mice (Arg1, IL-10). AscA infusion did not alter anti-inflammatory gene expression on day 5 although Arg1 levels were now similar to that observed in VitC sufficient mice (Figure 2(b), (B), (D), and (F)).

### 3.3. *Ex Vivo* Bacterial Lipopolysaccharide Differentially Activate Proinflammatory Gene Expression in Macrophages from VitC Sufficient and Deficient Mice

**Figure 3:**
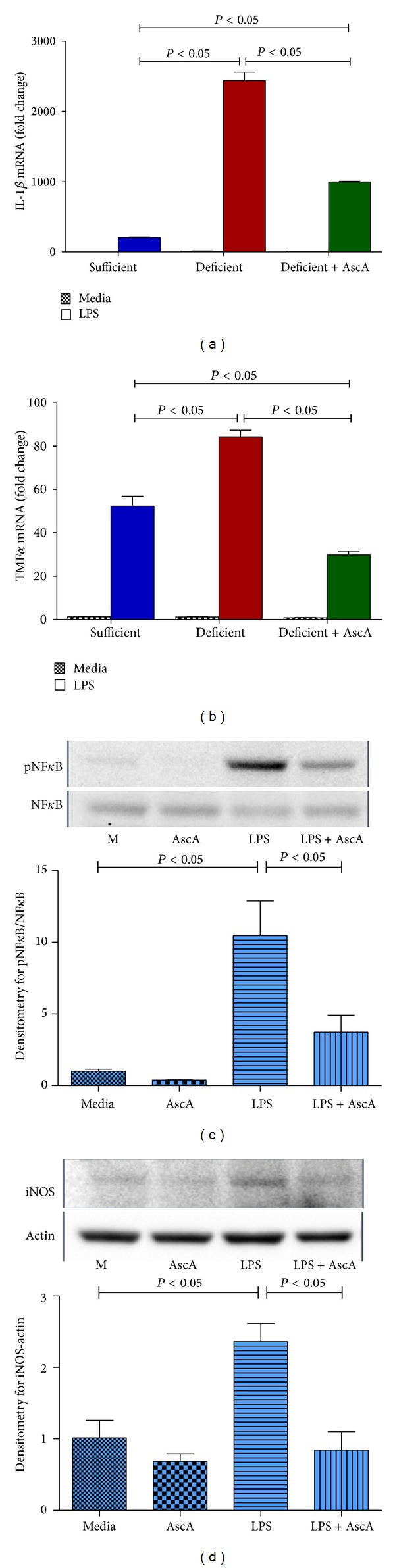
LPS differentially activates proinflammatory gene expression in macrophages from vitamin C sufficient and deficient mice. Peritoneal macrophages elicited on day 3 following TG-induced peritonitis from VitC sufficient and deficient Gulo^−/−^ mice were exposed to LPS (50 ng/mL) for 4 hours. Macrophages from some VitC deficient mice were incubated with AscA (3 mM, 16 hours) prior to LPS exposure (deficient + AscA). Real time QPCR was performed for IL-1*β* (a) and TNF*α* (b) (*N* = 6/group). (c) Upper panel: representative western blot for expression of phospho-NF*κ*B and NF*κ*B from VitC deficient macrophages exposed to media alone (M), AscA (3 mM, 16 hours (AscA)), LPS (50 ng/mL) for 1 hour (LPS), or AscA for 16 hours followed by LPS for 1 hour (LPS + AscA). Lower panel: densitometry for normalized expression of phospho-NF*κ*B from macrophages (*N* = 3/group). (d) Upper panel: representative western blot for expression of iNOS and actin from macrophages groups described in (c) and exposure to LPS (50 ng/mL) for 4 hour. Lower panel: densitometry for normalized expression of iNOS from macrophages (*N* = 3/group).

Canali et al. recently showed that in contrast with baseline physiological activation, exposure to a second “hit” such as an inflammatory stimulus results in a markedly different modulation of gene expression in human peripheral blood mononuclear cells in the presence or absence of VitC supplementation [45]. To examine whether peritoneal macrophages would exhibit an altered modulation of gene expression, we exposed day 3 peritoneal macrophages from VitC sufficient and deficient mice to bacterial lipopolysaccharide (LPS, 50 ng/mL). Some macrophages were incubated with AscA (3 mM, 16 hours) prior to LPS exposure. As seen in Figures 3(a) and 3(b), LPS exposure resulted in a robust increase in expression of proinflammatory markers (IL-1*β*, TNF*α*) in macrophages from VitC sufficient mice. Proinflammatory gene expression was also induced in macrophages from VitC deficient mice, but the magnitude of induction was significantly greater than that observed in the VitC sufficient macrophages (Figures 3(a) and 3(b)). Importantly, exposure of VitC deficient macrophages to AscA prior to LPS significantly attenuated IL-1*β* and TNF*α* expression. Increased NF*κ*B activation (Figure 3(c)) and iNOS protein expression (Figure 3(d)) was observed upon exposure of VitC deficient macrophages to LPS (*P* < 0.05). AscA pretreatment attenuated NF*κ*B activation and iNOS expression in VitC deficient macrophages.

### 3.4. VitC Regulates Macrophage Function during the Resolution of Inflammation

**Figure 4:**
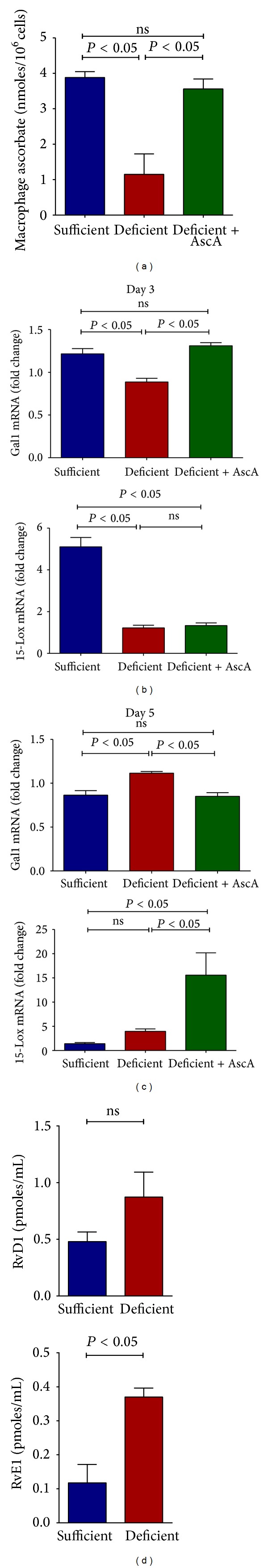
Vitamin C regulates macrophage function during the resolution of inflammation. (a) Macrophages were isolated on day 3 following TG-mediated peritonitis from VitC sufficient or deficient mice as well as in deficient mice treated daily  with i.p. AscA for 3 days and intracellular concentrations of VitC measured (*N* = 3–10 mice/group, ns = not significant). (b) Real time QPCR for Gal1 and 15-Lox from peritoneal macrophages elicited on day 3 following TG-induced peritonitis from VitC sufficient and deficient Gulo^−/−^ mice. Thirty minutes following TG challenge, some VitC deficient mice were randomized to receive i.p. injection of VitC as AscA (200 mg/kg in saline) for a further 3 days (deficient + AscA). (*N* = 6 mice/group, ns = not significant). (c) Real time QPCR for Gal1 and 15-Lox from peritoneal macrophages elicited on day 5 following TG-induced peritonitis from VitC sufficient and deficient Gulo^−/−^ mice. Thirty minutes following TG challenge, some VitC deficient mice were randomized to receive i.p. injection of VitC as AscA (200 mg/kg in saline) for a further 5 days (Deficient + AscA) (*N* = 6 mice/group, ns = not significant). (d) UPLC ESI-MS/MS quantification of resolvin D1 (RvD1) and E1 (RvE1) in peritoneal lavage on day 5 following TG-induced peritonitis from VitC sufficient and deficient Gulo^−/−^ mice (*N* = 3-4 mice/group, ns = not significant).

Macrophages undergo reprogramming to adopt a variety of functional phenotypes upon receiving differentiation cues from their surrounding environment [46]. It was recently shown that macrophage reprogramming is vital for resolution of acute inflammation [47]. We examined whether macrophage VitC sufficiency or deficiency could influence macrophage function during resolution of acute inflammation. Macrophages were isolated on day 3 following TG-mediated peritonitis from VitC sufficient or deficient mice and intracellular concentrations of VitC measured (as described in Section 2). Some VitC deficient mice were injected daily with i.p. AscA (200 mg/kg) prior to harvest of peritoneal lavage (see Section 2). As seen in Figure 4(a), macrophages from VitC sufficient mice have high intracellular VitC concentrations. In contrast, intracellular ascorbate levels were significantly depleted in macrophages from VitC deficient mice. Daily i.p. administration of AscA for 3 days also restored macrophage intracellular concentrations to levels observed in the VitC sufficient mice (Figure 4(a)).

Gal-1 and 15-Lox expression is induced in macrophages during peritonitis. Their expression is associated with generation of proresolving lipid mediators [11] and successful resolution of inflammation [48]. Therefore we examined Gal-1 and 15-Lox expression in day 3 and day 5 macrophages from VitC sufficient or deficient mice. As seen in Figure 4(b), Gal-1 and 15-Lox expression was significantly induced in macrophages from VitC sufficient mice on day 3 when compared to macrophages from VitC deficient mice. AscA infusion restored Gal-1 expression in VitC deficient macrophages but did not affect 15-Lox expression on day 3 (Figure 4(b)). In contrast, Gal-1 expression in VitC deficient macrophages was delayed and observed to be higher on day 5 following TG-induced peritonitis (Figure 4(c)). 15-Lox expression was induced by AscA infusion on day 5 and was higher than that observed in macrophages from VitC sufficient or deficient mice. In agreement with the expression data seen above, resolvin (Figure 4(d)) production was higher on day 5 in VitC deficient mice indicating delayed resolution of inflammation.

### 3.5. VitC Influences Macrophage Phenotype during Resolution of Inflammation

**Figure 5:**
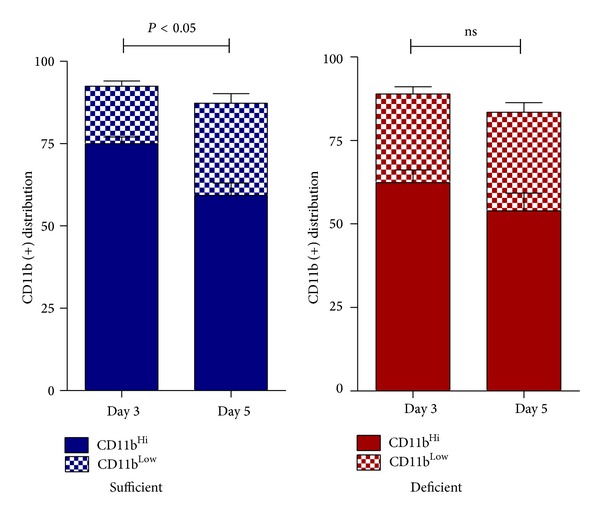
Vitamin C influences macrophage phenotype during resolution of inflammation. Flow cytometry for distribution of CD11b^high^ and CD11b^low^ population from macrophages isolated on day 3 and day 5 following TG-induced peritonitis in VitC sufficient or deficient mice (*N* = 5 mice/group, *P* < 0.05, CD11b^low^ day 3 versus day 5, ns = not significant).

Rostoker et al. recently showed that Gal-1 was selectively expressed in CD11b^high^ macrophages, and its expression declined significantly once these cells converted toward a CD11b^low^ phenotype [49]. Moreover, CD11b^low^ macrophages are the predominant subtype to depart the peritoneum [49]. To determine whether VitC regulated reprogramming of peritoneal macrophages to proresolution CD11b^low^ phenotype we used flow cytometry to examine the distribution of CD11b^high^ and CD11b^low^ population on macrophages isolated on day 3 and day 5 following TG-induced peritonitis in VitC sufficient or deficient mice. As seen in Figure 5, there was a significant transition from CD11b^high^ to a CD11b^low^ phenotype observed from day 3 to day 5 in the VitC sufficient macrophages. This was not evident in the macrophages from VitC deficient mice indicative of a delay in the resolution of TG-induced peritonitis in these mice.

### 3.6. Macrophages Deficient in VitC Have Reduced Antioxidant Capacity

**Figure 6:**
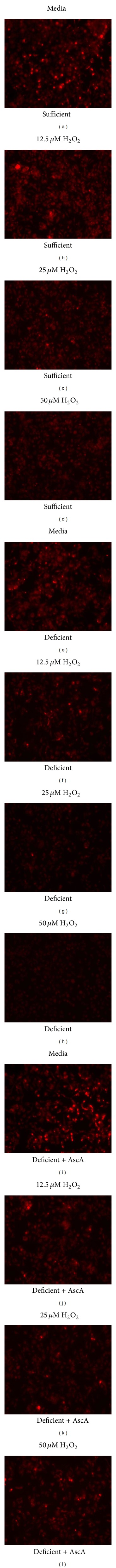
Macrophages deficient in Vitamin C have reduced antioxidant capacity. Peritoneal macrophages elicited on day 3 following TG-induced peritonitis from VitC sufficient ((a)–(d)) and deficient Gulo^−/−^ ((e)–(h)) mice were exposed to 12.5, 25, and 50 *μ*M H_2_O_2_ for 18 hours and probed with MitoTracker Red CMXRos. Macrophages from some VitC deficient mice were incubated with AscA (3 mM, 16 hours) prior to exposure to H_2_O_2_ followed by staining with MitoTracker Red CMXRos (Deficient + AscA, ((i)–(l))).

Activated macrophages potentially generate mitochondria-damaging deleterious reactive oxygen species (ROS). Release of large amounts of ROS during activation exposes macrophages themselves to oxidant stresses not encountered by most other cell types [50]. To test whether VitC deficiency affected mitochondrial function in macrophages, we exposed peritoneal macrophages (day 3) from VitC sufficient or deficient mice to varying concentrations of H_2_O_2_ for 18 hours and stained the cells with MitoTracker Red CMXRos as described in Section 2. This probe is selectively retained by mitochondria, where it is oxidized to its fluorescent form. As seen in Figures 6(a) and 6(e), control macrophages from VitC sufficient or deficient mice were stained brightly with the probe. Oxidative stress from exposure to H_2_O_2_ decreased fluorescent staining in macrophages from both VitC sufficient and VitC deficient mice. However, the magnitude of decrease was significantly greater in macrophages from VitC deficient mice (Figures 6(f)–6(h)). This decrease was partially reversed by pretreatment of VitC deficient macrophages with AscA (Figures 6(j) and 6(k)). These studies indicate that VitC deficient macrophages sustain greater mitochondrial dysfunction when challenged with ROS.

### 3.7. VitC Attenuates Proinflammatory Gene Expression in Human Monocyte/Macrophages

**Figure 7:**
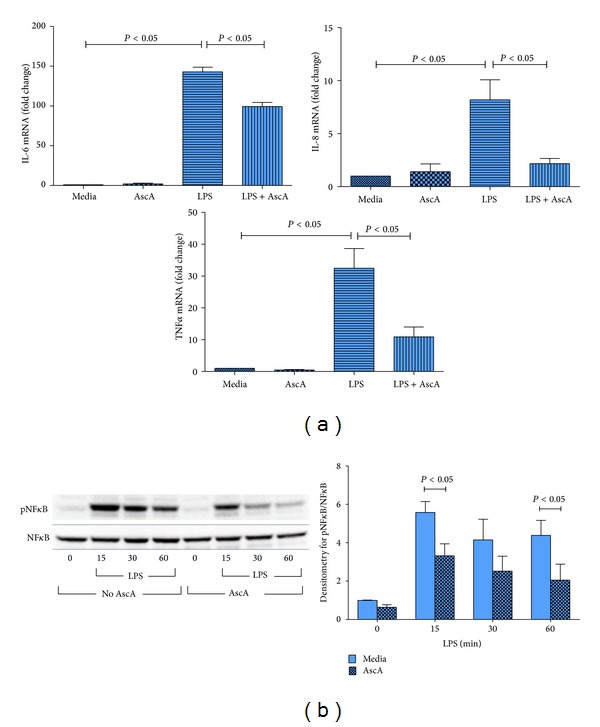
Vitamin C attenuates proinflammatory gene expression in human THP-1 monocyte/macrophages. THP-1 macrophages were exposed to media alone (Media), AscA (3 mM, 16 hours (AscA)), and LPS (50 ng/mL) for 4 hour (LPS) or AscA for 16 hours followed by LPS for 4 hour (LPS + AscA). (a) Real time QPCR for IL-6, IL-8, and TNF*α* was performed as described in Section 2 (*N* = 4/group; *P* < 0.05, Media versus LPS and LPS versus LPS + AscA). (b) Left panel: representative western blot for expression of phospho-NF*κ*B and NF*κ*B from THP-1 groups described above following exposure to LPS (50 ng/mL) for 0, 15, 30, and 60 minutes. Right panel: densitometry for normalized expression of phospho-NF*κ*B from THP-1 (*N* = 4/group, *P* < 0.05, LPS versus LPS + AscA).

To address whether the modulatory activities of VitC are effective in human monocyte/macrophages, we exposed THP-1 cells to bacterial LPS and examined the mRNA expression of the proinflammatory genes IL-6, IL-8, and TNF*α*. Since the culture medium in which THP-1 cells are grown contains no VitC, we increased intracellular concentrations of VitC by loading cells with AscA prior exposure to LPS. As seen in Figure 7(a), exposure of THP-1 cells to LPS resulted in a robust activation of mRNA for IL-6, IL-8, and TNF*α*. Loading cells with AscA did not affect baseline proinflammatory gene expression. However LPS exposure of AscA loaded cells resulted in significant attenuation of mRNA expression of these proinflammatory genes. Attenuation of mRNA expression was likely achieved by reduction in activation of the transcription factor NF*κ*B following LPS exposure (Figure 7(b)).

## 4. Discussion

In this study, we examined the mechanism by which VitC regulates the resolution of sterile inflammation. Using mice lacking the ability to synthesize VitC, we show that subnormal cellular VitC levels negatively impact the progression and resolution of sterile inflammation. In particular, our results demonstrate that low circulating VitC levels are associated with significant delays in the timing of resolution of inflammation. This apparent VitC-dependent process primarily occurs due to failure of macrophages to transition from a proinflammatory to a proresolving phenotype.

The initial response to sterile inflammation was identical in VitC sufficientanddeficient mice. During the early proinflammatory phase no differences in the cell numbers or cell types were observed. However, by days 3 and 5, VitC deficient mice exhibited significant numbers of PMN in peritoneal exudates (Table 2). Spatiotemporal mRNA profiling of macrophage-derived inflammatory mediators revealed dramatic differences in the magnitude of pro- and anti-inflammatory mediator gene expression (Figure 2). Macrophages from VitC sufficient mice displayed prominent anti-inflammatory phenotypes, while VitC deficient macrophages persistently expressed mRNA for IL-1*β*, TNF*α*, and MCP-1, findings characteristic of a proinflammatory phenotype. LPS activation of day 3 macrophages from VitC deficient mice led to proinflammatory gene expression that was significantly greater in magnitude than that observed in VitC loaded macrophages (Figure 3). LPS stimulation was characterized by enhanced NF*κ*B activation and iNOS induction in VitC deficient macrophages (Figure 3). Importantly, on day 3, VitC sufficient macrophages demonstrated cues for reprogramming into resolution type macrophages, a vital step required for resolution of inflammation. In day 3 macrophages from VitC sufficient mice, expression of Gal-1 and 15-Lox mRNA was robust (Figure 4). In contradistinction, enhanced Gal-1 and 15-Lox mRNA expression was delayed to day 5 in VitC deficient macrophages. The delays in resolution we observed in VitC deficient mice were confirmed by quantification of resolvins in peritoneal exudates; increases of which were present only on day 5 (Figure 4). Further confirmation of altered spatiotemporal relationships was achieved by studying macrophage phenotypic changes by examining the distribution of CD11b on macrophages from VitC sufficient or deficient mice on day 3 and day 5 following TG-induced peritonitis (Figure 5). Phenotypic changes in macrophages were accompanied by alterations in macrophage function as demonstrated by the increased susceptibility of VitC deficient macrophages to mitochondrial dysfunction when exposed to reactive oxygen species (Figure 6). In final studies, we employed the human monocyte/macrophage cell line THP-1, which lacks VitC in culture medium when cultured under standard conditions. We demonstrated increased proinflammatory gene expression in THP-1 when exposed to LPS under VitC-deprived conditions. Loading THP-1 cells with AscA significantly attenuated mRNA expression of proinflammatory genes via a mechanism likely involving reduced activation of the transcription factor NF*κ*B. VitC loading was effective both* in vitro* and* in vivo* since daily AscA infusion following induction of peritonitis significantly restored macrophage phenotype and function in the VitC deficient mice.

Few studies have examined the role of VitC in resolution of sterile inflammation. Ganguly et al. initially reported that VitC deficiency affected migration of guinea pig macrophages under* in vitro* conditions [51]. They further showed that addition of exogenous VitC partially restored the migratory response. May et al. showed that activated macrophages use ascorbate to lessen self-generated oxidant stress [18]. They later showed that ascorbate deficient peritoneal macrophages were more susceptible to H_2_O_2_-induced mitochondrial dysfunction and apoptosis [52]. However no studies to date have examined macrophage function during resolution of inflammation in mice lacking the ability to synthesize their own VitC. Our observation of persistence of PMN at the site of inflammation in VitC deficient mice is in agreement with our previous results [28] and those of Vissers and Wilkie who used a similar TG model of peritonitis to show impairment in PMN apoptosis and clearance [19]. It has been suggested that the engulfment of apoptotic cells is generally anti-inflammatory or immunologically silent [53] due to the fact that it sequesters dying cells thus preventing release of potentially toxic cell contents into the local environment. Based on the observations that PMN persists for up to 5 days in the peritoneum of VitC deficient mice (Table 2), it is therefore possible that the apoptosis-resistant PMN can cause strong proinflammatory responses from the macrophages that extravasate to sites of inflammation. Indeed strong and persistent proinflammatory responses were evident in VitC deficient macrophages elicited on day 3 and even day 5 (Figure 2).

Efferocytosis, a process by which dead and/or dying cells are being engulfed and removed by other cells, has been reported to induce production of anti-inflammatory mediators from macrophages that suppress inflammation thereby silently clearing apoptotic cells and thus dampening proinflammatory responses [54]. VitC sufficient mice exhibited anti-inflammatory mediator expression in macrophages early (day 3) in the post TG-induced inflammatory process, a phenomenon indicative of functional efferocytosis. In contrast, VitC deficient macrophages failed to upregulate anti-inflammatory mediator production until day 5 (Figure 2).

Gal-1 and 12/15-Lox play vital roles in resolution of inflammation. Rostoker et al. have shown that Gal-1 promotes the generation of M2-like macrophages, which then favors tissue repair during early resolution of inflammation [49]. Ariel and Timor demonstrated that Gal-1 promotes generation of M_res_ from M2 macrophages, which generates proresolving lipid mediators. This phenotype change promotes macrophage departure from peritoneal cavities with resolving inflammation, thus allowing return of tissue to homeostasis [55]. Moreover, Gal-1 expression, which is enhanced in CD11b^high^ macrophages, declines sharply as cells revert to the CD11b^low^ phenotype. CD11b^low^ macrophage phenotypes, as noted previously, promote departure from peritoneal cavities with resolving inflammation [49]. Our findings (Figures 4 and 5) which agree with the above studies implicate VitC as a critical regulator of macrophage transition during resolution of inflammation. Expression and function of 12/15-Lox produce key mediators (e.g., lipoxins, resolvins, protectins, and maresins) that promote resolution of proinflammatory pathologies [56]. In particular, human and murine monocytes/macrophages expression of 15-Lox is upregulated by efferocytosis with production of mediators such as RvD1, a mediator shown to promote the resolution of murine peritonitis [47, 57]. Further, Gal-1 directly promotes 15-lipoxygenase expression and activity in macrophages during the inflammatory and resolving phases of peritonitis [49]. The earlier increases in Gal-1 and 15-Lox mRNA expression in VitC sufficient macrophages (Figures 4(b) and 4(c)) and the delayed resolvin production in the VitC deficient macrophages (Figure 4(d)) indicate for the first time that VitC influences multiple processes leading to the resolution of inflammation.

## 5. Conclusions

The findings in this mouse model have significant human relevance since VitC levels are subnormal in multiple human inflammatory disease states including sepsis, systemic inflammatory response syndrome (SIRS), trauma, and cancer, among others. In a recently completed Phase I trial (ClinicalTrials.gov identifier NCT01434121) of intravenous AscA in critically ill patients with severe sepsis, we showed that septic patients exhibited abnormally low VitC plasma levels and that intravenous AscA infusion could significantly increase circulating VitC levels [29]. Further, AscA infusion significantly reduced the proinflammatory biomarkers C-reactive protein and procalcitonin as well as multiple organ dysfunction [29]. Our findings here add a previously unrecognized element to our understanding of the machinery that governs the resolution of inflammation.

## Figures and Tables

**Table 1:** Primer sequences for real time quantitative PCR.

Name	Sequences 5′ to 3′
Murine IL-1*β* forward	CTGAACTCAACTGTGAAATGCC
Murine IL-1*β* reverse	CAGGTCAAAGGTTTGGAAGC

Murine TNF*α* forward	GATGAGAAGTTCCCAAATGGC
Murine TNF*α* reverse	TTGGTGGTTTGCTACGACG

Murine MCP-1 forward	TTCTGGGCCTGCTGTTCACAG
Murine MCP-1 reverse	CCAGCCTACTCATTGGGATCATCTTGC

Murine YM1 forward	CAAGACTTGCGTGACTATGAAGC
Murine YM1 reverse	AGGTCCAAACTTCCATCCTCC

Murine Arg1 forward	AGGAAAGCTGGTCTGCTGG
Murine Arg1 reverse	TTGAAAGGAGCTGTCATTAGGG

Murine IL-10 forward	CAAGGAGCATTTGAATTCCC
Murine IL-10 reverse	ATTCATGGCCTTGTAGACACC

Murine Gal1 forward	CAGCAACCTGAATCTCAAACC
Murine Gal1 reverse	AGTGTAGGCACAGGTTGTTGC

Murine 15-Lox forward	TGGTGGCTGAGGTCTTTGC
Murine 15-Lox reverse	TCTCTGAGATCAGGTCGCTCC

Human IL-6 forward	GGATTCAATGAGGAGACTTGCC
Human IL-6 reverse	TCTGCAGGAACTGGATCAGG

Human IL-8 forward	GTGTGAAGGTGCAGTTTTGC
Human IL-8 reverse	GAGCTCTCTTCCATCAGAAAGC

Human TNF*α* forward	CCTCTTCTCCTTCCTGATCG
Human TNF*α* reverse	CGAGAAGATGATCTGACTGCC

**Table 2:** Differential cell counts from peritoneal exudates following thioglycollate-induced peritonitis (*N* = 6–8 mice/group, n.d. = not determined).

		Sufficient	Deficient	Deficient + AscA
Day 1	PMN (×10^6^)	20.02 ± 3.4	23.2 ± 2.6	n.d.
	M0 (×10^6^)	5.0 ± 0.8	3.9 ± 0.6	n.d.

Day 3	PMN (×10^6^)	1.8 ± 0.4	8.1 ± 1.9^a^	3.8 ± 0.4
	M0 (×10^6^)	18.4 ± 3.2	13.1 ± 3.1	14.5 ± 3.2

Day 5	PMN (×10^6^)	1.0 ± 0.6	4.4 ± 1.1^b^	0.4 ± 0.2^c^
	M0 (×10^6^)	18.8 ± 5.6	22.1 ± 4.2	14.3 ± 4.1

^a^Sufficient versus deficient, *P* = 0.006.

^b^Sufficient versus deficient, *P* = 0.02.

^c^Deficient versus deficient + AscA, *P* = 0.02.
